# Effect of Oxygen Contamination on Propionate and Caproate Formation in Anaerobic Fermentation

**DOI:** 10.3389/fbioe.2021.725443

**Published:** 2021-09-10

**Authors:** Flávio C. F. Baleeiro, Magda S. Ardila, Sabine Kleinsteuber, Heike Sträuber

**Affiliations:** ^1^ Department of Environmental Microbiology, Helmholtz Centre for Environmental Research – UFZ, Leipzig, Germany; ^2^ Institute of Process Engineering in Life Science 2, Technical Biology, Karlsruhe Institute of Technology – KIT, Karlsruhe, Germany

**Keywords:** carboxylate platform, lactate-based chain elongation, mixotrophy, micro-aerobic fermentation, open mixed culture, caproic acid, propionic acid, gas recirculation

## Abstract

Mixed microbial cultures have become a preferred choice of biocatalyst for chain elongation systems due to their ability to convert complex substrates into medium-chain carboxylates. However, the complexity of the effects of process parameters on the microbial metabolic networks is a drawback that makes the task of optimizing product selectivity challenging. Here, we studied the effects of small air contaminations on the microbial community dynamics and the product formation in anaerobic bioreactors fed with lactate, acetate and H_2_/CO_2_. Two stirred tank reactors and two bubble column reactors were operated with H_2_/CO_2_ gas recirculation for 139 and 116 days, respectively, at pH 6.0 and 32°C with a hydraulic retention time of 14 days. One reactor of each type had periods with air contamination (between 97 ± 28 and 474 ± 33 mL O_2_ L^−1^ d^−1^, lasting from 4 to 32 days), while the control reactors were kept anoxic. During air contamination, production of *n*-caproate and CH_4_ was strongly inhibited, whereas no clear effect on *n*-butyrate production was observed. In a period with detectable O_2_ concentrations that went up to 18%, facultative anaerobes of the genus *Rummeliibacillus* became predominant and only *n*-butyrate was produced. However, at low air contamination rates and with O_2_ below the detection level, Coriobacteriia and Actinobacteria gained a competitive advantage over Clostridia and Methanobacteria, and propionate production rates increased to 0.8–1.8 mmol L^−1^ d^−1^ depending on the reactor (control reactors 0.1–0.8 mmol L^−1^ d^−1^). Moreover, *i-*butyrate production was observed, but only when Methanobacteria abundances were low and, consequently, H_2_ availability was high. After air contamination stopped completely, production of *n*-caproate and CH_4_ recovered, with *n*-caproate production rates of 1.4–1.8 mmol L^−1^ d^−1^ (control 0.7–2.1 mmol L^−1^ d^−1^). The results underline the importance of keeping strictly anaerobic conditions in fermenters when consistent *n*-caproate production is the goal. Beyond that, micro-aeration should be further tested as a controllable process parameter to shape the reactor microbiome. When odd-chain carboxylates are desired, further studies can develop strategies for their targeted production by applying micro-aerobic conditions.

## Introduction

Anaerobic fermentation with mixed microbial communities is an appealing option for the production of medium-chain carboxylates (MCCs) (De Groof et al., 2019). However, the high degree of complexity of mixed communities is an additional obstacle to achieve a stable and feasible bioprocess. Changes in operation parameters favor some microorganisms while negatively affecting others in ways that are hard to predict. Controlled experiments with defined substrates can help to understand the response of microbial networks to disturbances and to develop more robust fermentation processes (Angenent et al., 2016; Andersen et al., 2017; Oleskowicz-Popiel, 2018).

Oxygen from the air can easily enter anaerobic reactors by diffusion due to incomplete tightness or oxic feedstocks, and is considered a disturbance of the anaerobic processes. At first thought, MCC-producing mixed cultures should be protected from O_2_ at all costs. So far, almost all isolated MCC producers are strict anaerobes (one exception was described by Stamatopoulou et al. (2020)) to which O_2_ causes damage *via* direct and indirect ways. O_2_ gives rise to reactive oxygen species (ROS), such as O_2_^−^ and H_2_O_2_, which are intermediates produced during O_2_ reduction that severely damage cells if not promptly neutralized (Johnson and Hug, 2019). Even though every cultured microorganism has mechanisms to deal with ROS (Johnson and Hug, 2019), obligate anaerobic bacteria such as *Clostridium* spp. suffer particularly from O_2_ due to their high dependence on O_2_-sensitive enzymes (e.g., ferredoxin-dependent oxidoreductases or [FeFe]-hydrogenases) (Imlay, 2006; Khademian and Imlay, 2020). Most hydrogenases, which also contain Fe-S clusters, are reversibly or irreversibly inhibited by O_2_ and its activated forms. Exposure to O_2_ causes some hydrogenases to decompose or to form additional ROS that damage other parts of the cell (Stiebritz and Reiher, 2012).

Oxygen contamination does not necessarily mean a complete failure of the anaerobic process and its effect depends on the contamination rate and on the ability of the anaerobic community to remove the contaminant (Botheju and Bakke, 2011). Facultative anaerobic microorganisms present in mixed cultures can consume traces of oxygen and thus protect strict anaerobes (Nguyen and Khanal, 2018). As an exemplary proof of concept, the facultative anaerobe *Parageobacillus thermoglucosidasius* has been used for O_2_ scrubbing before feeding waste gases to acetogenic cultures (Mohr et al., 2019). Additionally, the presence of biofilms, microbial aggregates, and other types of diffusion gradients in reactors can form protective oxygen barriers (Botheju and Bakke, 2011).

Uncontrolled aeration during anaerobic fermentation, e.g., *via* the supply of oxic substrates, can lead to the presence of strict aerobic microorganisms (Lambrecht et al., 2019). On the other hand, small amounts of oxygen may be desired in anaerobic processes. Micro-aeration, the controlled dosing of small amounts of air or oxygen (loosely defined from 5 to 5,000 mL O_2_ L^−1^ d^−1^), has been mainly used to create different oxidative-reductive regions in digesters to favor biological desulfurization (Krayzelova et al., 2015; Nguyen and Khanal, 2018). Besides, it has been reported that micro-aeration can enhance the hydrolysis step in anaerobic digestion by increasing the production of extracellular enzymes such as proteases, amylases, and cellulases (Girotto et al., 2016). Presence of O_2_ can also be advantageous in fermentations with acetogens. During batch cultivation of *Clostridium ljungdahlii* on H_2_, CO, CO_2_, and fructose, 8% O_2_ in the headspace has been found to increase the production of ethanol (Whitham et al., 2015), which is an electron donor for chain elongation.

Although the possibilities with O_2_ are being explored in many types of anaerobic technologies, no literature can be found about the effects of controlled O_2_ contamination rates on chain elongation systems. One possible reason is that designing controlled experiments to understand the effects of small oxygen contamination in mixed microbial communities is not trivial. Distribution and monitoring of O_2_ can be challenging at the low concentrations found in micro-aerated systems (Nguyen and Khanal, 2018). Recently, an anaerobic reactor system with continuous gas recirculation was presented as a way to ensure high gas availability to the microbial community at all times, while keeping the system closed and allowing to track component balances in the gas phase (Baleeiro et al., 2021b).

With the help of gas recirculation systems, this study aimed to investigate the effects of small air contamination on the dynamics of the microbial community and the product formation in MCC-producing fermenters. For this purpose, two stirred tank reactors (STRs) and two bubble column reactors (BCRs) with continuous H_2_/CO_2_ recirculation were operated with mixed cultures fed with lactate and acetate. One of the reactors of each type had periods with air contamination, while the other two were kept anoxic.

## Materials and Methods

### Experimental Design and Reactor Systems

Two pairs of reactors with continuous gas recirculation were assembled for this study. One pair consisted of stirred tank reactors (STR-control and STR-test) and the other of bubble column reactors (BCR-control and BCR-test). Assembly and configuration of the STRs were described in detail by Baleeiro et al. (2021b). BCRs were assembled and operated with the following differences in relation to the STRs: 1) the BCR vessels consisted of bubble columns made of glass with a working volume of 1.2 L each; 2) the systems had no oxidation-reduction potential (ORP) monitoring; 3) pH monitoring and correction was done manually three times a week; 4) temperature regulation was carried out *via* the water jacket of the vessels and a thermal bath; 5) gas recirculation was carried out with micro-diaphragm gas pumps NMP 830 (KNF Neuberger GmbH, Freiburg, Germany) at a flow of ca. 1.5 L min^−1^; and 6) an internal vertical hollow glass with holes of 1–2 mm was used to bubble the gas into the broth. In all other aspects, BCRs were operated similarly to STRs. The reactors were operated at 32°C, at pH values of 6.0 ± 0.1 (STRs) and 6.1 ± 0.3 (BCRs), and with a hydraulic retention time (HRT) of 14 days. The basal medium contained 133 mM lactate (12 g L^−1^) and 200 mM acetate (12 g L^−1^) as organic carbon sources and the gas reservoirs were periodically refilled with 10 L H_2_:CO_2_ (80:20), 240 mL ethylene (methanogenesis inhibitor), and 120 mL He (tracer gas). Basal medium composition, reactor start-up and reactor operation were identical for STRs and BCRs and were as described by Baleeiro et al. (2021b), except that lactate was fed ten times per day along with the basal medium.

**FIGURE 1:**
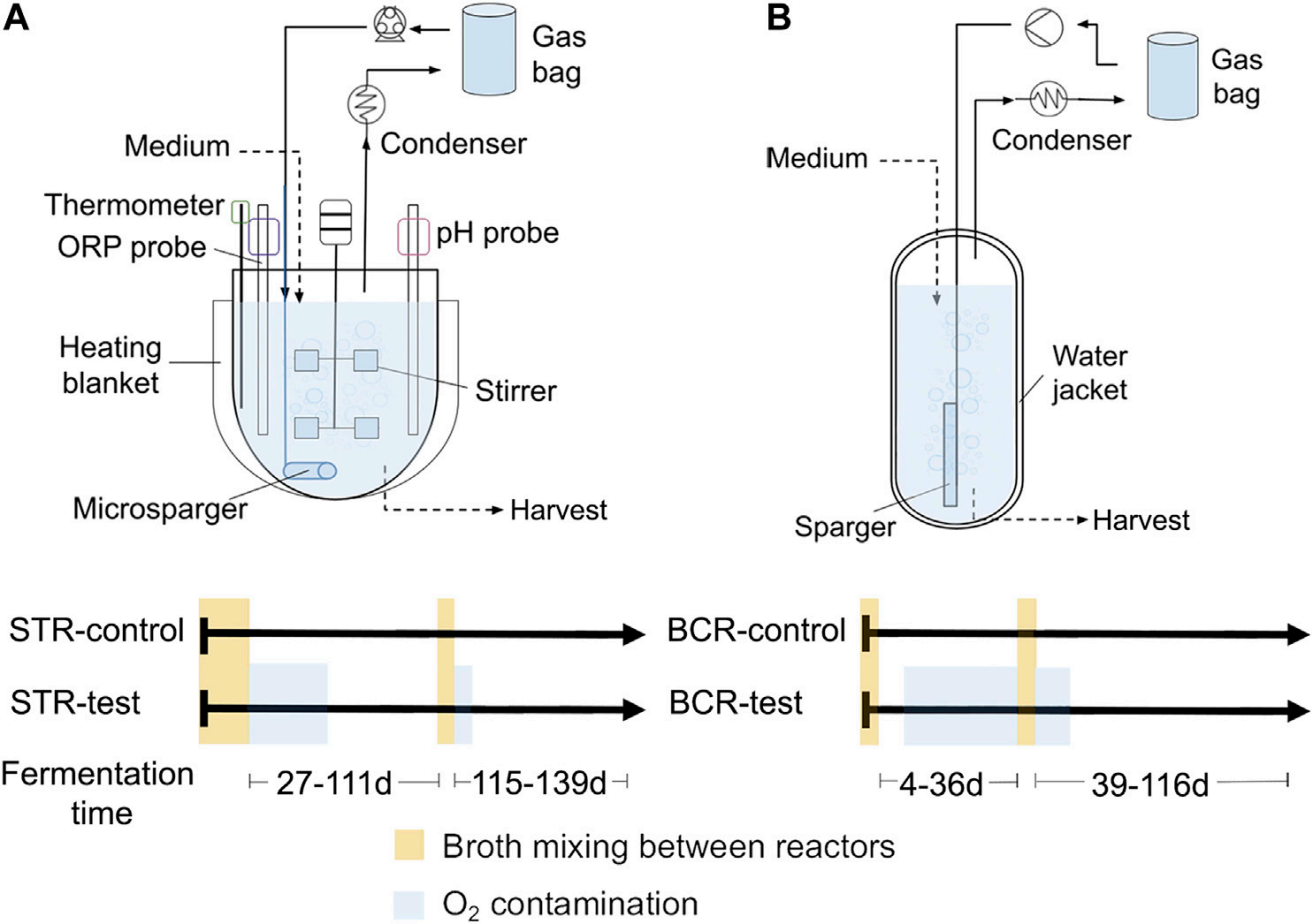
Gas recirculation reactors used in the study. Stirred tank reactors (STRs) **(A)** and bubble column reactors (BCRs) **(B)** with their respective timelines of events for each reactor.

Figure 1 shows the two reactor types used in the study and the timelines with the oxygen contamination events. The four reactors were inoculated with the broth harvested from the H_2_/CO_2_/ethylene recirculation reactors described by Baleeiro et al. (2021b). For STRs, the broths of the two reactors were mixed before the start of the experiment and before the second comparison period to ensure that both reactors started with identical microbial and chemical compositions for each comparison period. The same was done with the broths of the BCRs. Startup and broth mixing of STR-test and STR-control occurred on operation days 0–27 and the second broth mixture occurred on days 111–115 (Figure 1A). Startup and acclimatization of the community to a BCR occurred during the 86 days preceding the start of this study. Mixing of the BCR-control and BCR-test broths occurred on days 0–4 and 36–39 (Figure 1B). The broths were mixed without opening the reactors using Hei-Flow Precision peristaltic pumps (Heidolph Instruments GmbH, Schwabach, Germany), PVC tubes Tygon^®^ LMT-55, and three-way valves. The major air contamination events were detected in STR-test on days 27–59 and 115–119 and in BCR-test on days 11–36 and 39–50 (Figure 1).

The reactors that remained air-tight all the time were used as control reactors. Reactors STR-test and BCR-test presented detectable air intrusion at certain periods due to imperfect air-tight conditions. With the help of a H_2_ leak detector (GLD-100, Coy Laboratory Products, Grass Lake, USA), tightness problems that caused gas to leak out of the reactor systems were located and promptly solved. The same was not true for tightness problems that only allowed air to leak into the system and that were solved by trial and error interventions.

### Analytical Methods

High performance liquid chromatography with a refractive index detector (HPLC Prominence-i RID, Shimadzu Europa GmbH, Duisburg, Germany) was operated under the conditions described by Apelt (2020) with adaptations described by Baleeiro et al. (2021a) for monitoring of the following chemicals in the aqueous phase: formate, acetate, ethanol, lactate, propionate, *n*-propanol, *n*-butyrate, *i*-butyrate, *n-*butanol, *n-*valerate, *i-*valerate, *n-*pentanol, *n-*caproate, *i-*caproate, *n-*hexanol, *n-*heptanoate, and *n-*caprylate. Biomass concentration was determined by measuring optical density at 600 nm (spectrophotometer Genesys 10 S, Thermo Scientific Inc., Waltham, United States). Conversion factors for optical density at 600 nm and biomass are described previously (Baleeiro et al., 2021a). In the gas phase, H_2_, CO_2_, CH_4_, He, O_2_, N_2_, and ethylene were monitored by gas chromatography as described by Logroño et al. (2020).

### Microbial Community Analysis

Microbial communities were analyzed by 16S rRNA gene amplicon sequencing with taxonomic assignments done with the SILVA 138 reference database (Yilmaz et al., 2014). DNA extraction, PCR, and library preparation for Illumina MiSeq sequencing were described previously (Baleeiro et al., 2021a). Trimming, filtering, and denoising of amplicon data as well as visualization of microbiome census data and Spearman correlations were done as described by Baleeiro et al. (2021a). All samples were rarified to an equal depth according to the sample with the lowest read number in the dataset (4,977 counts). Raw sequence data for this study was deposited at the European Nucleotide Archive (ENA) under the study accession number PRJEB44209 (http://www.ebi.ac.uk/ena/data/view/PRJEB44209).

### Component Balance and Estimation of O_2_ Contamination

Assumptions and calculation steps used for the component balances in the gas recirculation reactors were described previously (Baleeiro et al., 2021b). Direct measurements of O_2_ concentration (such as by using dissolved oxygen probes) are not adequate to monitor micro-aerobic environments (Krayzelova et al., 2015). Therefore, O_2_ contamination was determined indirectly *via* the N_2_ concentration in the recirculating gas, using the N_2_:O_2_ ratio in air of 3.73 according to the following equation:

O2 contamination rate [mL d−1]= (Y2Vgas 2−Y1Vgas 1)[3.73 (t2−t1)] (1)

for sampling points 1 and 2: *Y* is the volumetric fraction of N_2_ in the gas phase; *V*_*gas*_ is the gas volume of the system in mL; and *t* is the sampling time in days. Eq. 1 holds true if no N_2_ is formed or consumed in the reactor, if O_2_ is below the detection limit, and if the increase in the system’s gas volume due to air leaking in can be neglected. Calculations of contamination rates in the control reactors (STR-control and BCR-control) were used to determine standard errors for the O_2_ contamination rate (Supplementary Figure S1). ORP measurements did not show a clear relation with O_2_ contamination rates (Supplementary Figure S2) and were, hence, not used to quantify rates.

## Results

The experiments were divided into two periods, each with one air contamination event. STR-test and BCR-test were contaminated with O_2_ from air for certain periods, while STR-control and BCR-control remained virtually anoxic throughout the study (calculated average contamination rates of 7 ± 33 and 3 ± 28 mL O_2_ L^−1^ d^−1^, respectively) and were adopted as controls for anoxic reactor operation. Before the start of each period, the broths of each reactor pair were mixed so that each pair started with a similar microbial community and broth composition. The broths from the STRs were not mixed with the broths from the BCRs, resulting in communities developing independently for each reactor type.

### General Performance of the Gas Recirculation Reactors

The main carboxylates produced were propionate, *n*-butyrate, *i*-butyrate, *n-*valerate, *n*-caproate, and *n*-caprylate, with maximum concentrations presented in Table 1. The STRs reached higher *n*-caproate maxima and were the only ones in which *n*-caprylate was detected, whereas the BCRs reached higher propionate, *n-*valerate, and *i-*butyrate maxima. These differences can also be seen in terms of specific production and consumption rates given in mmol L^−1^ d^−1^ in Supplementary Table S1. For all reactors, most of the lactate fed was consumed and differences in its consumption rates were due to washout of unconsumed substrate (Supplementary Table S1). Except for the period when O_2_ was present in detectable amounts, net consumption of acetate occurred in all reactors, ranging from 1.4 to 4.4 mmol L^−1^ d^−1^, which corresponded to a small fraction of the total acetate fed (14.3 mmol L^−1^ d^−1^). With the exception of STR-control, where very little *i*-butyrate was produced, *i*-butyrate production rates ranged from 0.23 to 1.08 mmol L^−1^ d^−1^ and showed no clear relation to O_2_ contamination (Supplementary Table S1).

**TABLE 1:** Maximum concentration of carboxylates achieved in the experiments.

Reactor	Maximum concentration (g L^−1^)					
	propionate	*n*-butyrate	*i*-butyrate	*n*-valerate	*n*-caproate	*n*-caprylate
STR-control	1.3	3.4	0.5	0.3	4.6	1.1
STR-test	1.7	5.7	0.6	0.4	3.5	1.0
BCR-control	2.7	4.1	2.2	1.0	3.0	—
BCR-test	3.1	4.7	1.6	0.6	3.5	—

According to previous experience with the H_2_/CO_2_ recirculation reactors, ethylene was used to inhibit CH_4_ production. Even though the partial pressure of ethylene was higher than 1 kPa at all times, methanogens gradually acclimatized to the inhibitor. Methanogenesis was observed first in the reactors that remained anoxic throughout the experimental time: in BCR-control from day 0 and in STR-control from day 31. Later on, methanogenesis was also observed in STR-test from day 48 and in BCR-test from day 60. Methane production rates were similar in the control reactors STR-control and BCR-control (16.5 and 15.9 mmol L^−1^ d^−1^, respectively) and the highest rate observed over a sustained period was 19.5 mmol L^−1^ d^−1^ during an anoxic operation period between days 59 and 111 of STR-test (Supplementary Table S1).

With 200 mM acetate (12 g L^−1^) originally present in the growth medium, no net acetate formation was observed and no clear signs of homoacetogenic activity were found. After discounting the H_2_ used for CH_4_ formation (assuming 1 mol CH_4_ produced from 4 mol H_2_), almost no additional H_2_ consumption was seen in the control reactors STR-control and BCR-control. STR-control had a net H_2_ formation of 3.73 mmol L^−1^ d^−1^, whereas BCR-control showed a net H_2_ consumption of 0.50 mmol L^−1^ d^−1^ (Supplementary Table S1). In the periods with O_2_ contamination, H_2_ consumption rates remained relatively high, despite low methanogenic activity. STR-test showed additional H_2_ consumption of 26.3 mmol L^−1^ d^−1^ between days 27 and 59 and of 64.7 mmol L^−1^ d^−1^ between days 115 and 119 (Supplementary Table S1). This consumption of H_2_ corresponded from 3.0 to 3.4 times the molar consumption of O_2_ in the same period. In aerated periods in BCR-test, H_2_ consumption after discounting methane production ranged from 1.1 to 3.6 times the oxygen consumption.

Electron balances encompassing the whole period of fermentation had errors of -0.63% (i.e., 0.63% of the monitored pool of electron equivalents had unexplained consumption) for STR-test, −0.73% for STR-control, −0.93% for BCR-control, and −1.61% for BCR-test.

### Effect of O_2_ on the Fermentation in the Stirred Tank Reactor

**FIGURE 2:**
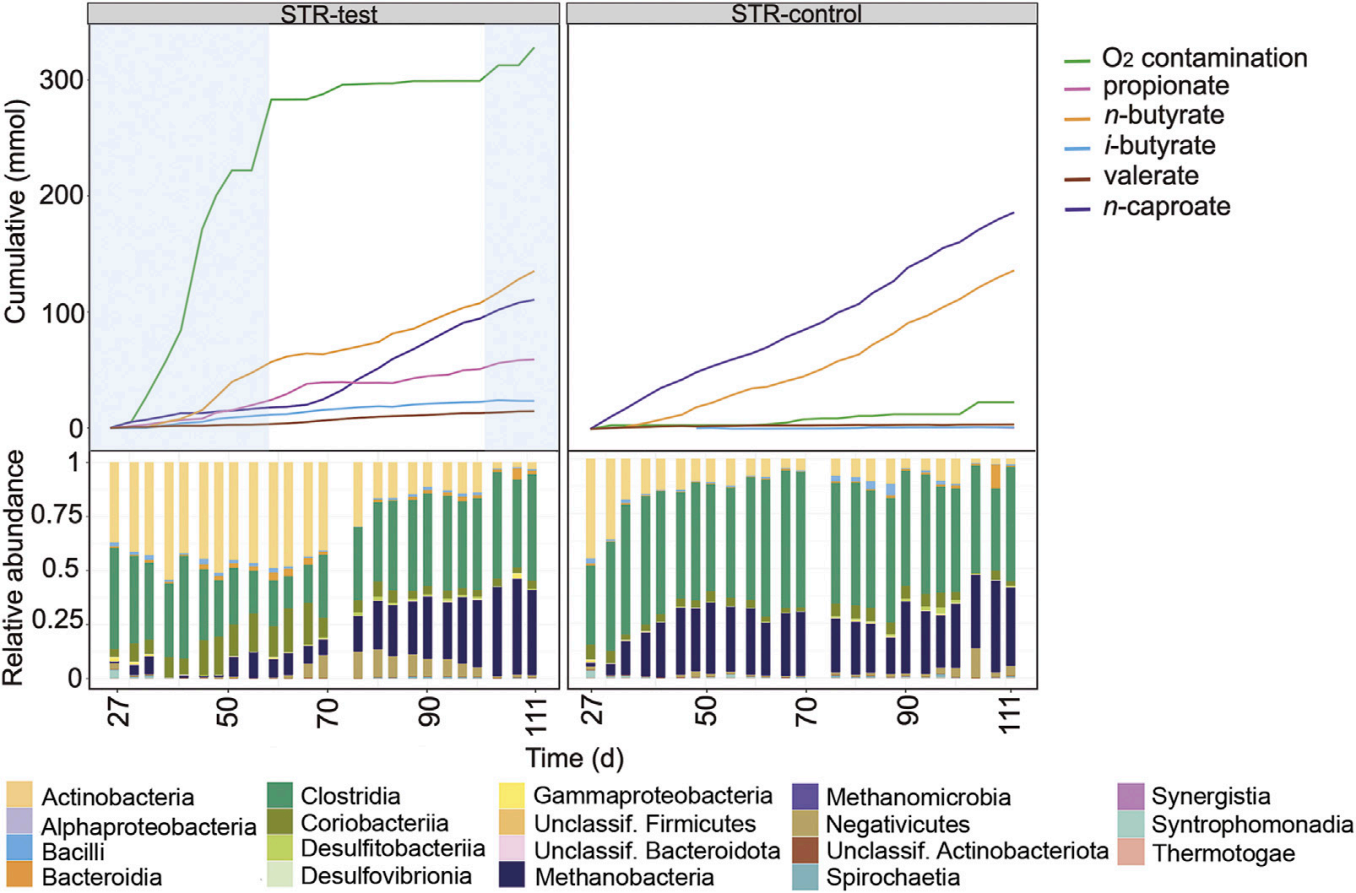
Profiles of the cumulated amounts of carboxylates and O_2_, as well as community composition at class level between days 27 and 111 for STR-test and STR-control. Blue shading indicates the O_2_ contamination period.

Figure 2 shows the profiles of the cumulated amounts of carboxylates and O_2_ as well as the microbial community composition at class level for the first period comparing the STRs. Between days 27 and 59, O_2_ concentration in the gas phase remained below the detection limit in STR-test (Supplementary Figure S3), although a contamination rate of 220 ± 33 mL O_2_ L^−1^ d^−1^ was detected. Between days 59 and 111, STR-test had an anoxic operation period although small O_2_ contaminations occurred between days 104 and 111 (Figure 2) and were the reason why the reactor could not reach perfectly anoxic conditions (21 ± 33 mL O_2_ L^−1^ d^−1^).

Even though *n*-butyrate and *n*-caproate were the main carboxylates produced in both reactors, *n*-caproate production in STR-test was, with 0.56 mmol L^−1^ d^−1^, 72% lower than in STR-control (2.12 mmol L^−1^ d^−1^) during the contamination period (days 27–59, Figure 2). Under O_2_ stress, STR-test produced more propionate (0.76 mmol L^−1^ d^−1^, 6.4 times that of the control) but *n*-butyrate production was similar in both reactors. Moreover, O_2_ contamination in STR-test caused 63% less methane production (6.05 of 16.5 mmol L^−1^ d^−1^) and 38% less acetate consumption (1.80 of 2.91 mmol L^−1^ d^−1^, Supplementary Table S1). After anoxic conditions in STR-test had been restored (days 59–111), methane production was 18% higher than in the control reactor (19.5 of 16.5 mmol L^−1^ d^−1^) and propionate production decreased slightly to 0.67 mmol L^−1^ d^−1^. Under anoxic conditions in STR-test, propionate production slowed down from day 66 on, coinciding with an acceleration of *n*-caproate production (Figure 2), which was still 16% lower than in the control (1.78 of 2.12 mmol L^−1^ d^−1^).

O_2_ contamination caused differences in microbial community composition that were visible up to the class level (Figure 2). Clostridia and Methanobacteria predominated in the reactor that remained completely anoxic. In the other reactor, Actinobacteria and Coriobacteriia were the main classes found in periods when O_2_ contamination occurred. The community of STR-test converged to a composition similar to that of the control reactor after anoxic conditions had been restored.

For the same operation period, community composition with resolution to the genus level and concentrations of the main carboxylates are shown in Supplementary Figure S4. Clostridia were mainly represented by the genera *Caproiciproducens*, *Clostridium sensu stricto* 12, and *Oscillibacter,* while the Actinobacteria belonged to the genera *Acidipropionibacterium* and *Actinomyces*. Methanogens were from the genus *Methanobrevibacter* and the Coriobacteriia were from the family *Eggerthellaceae*.

**FIGURE 3:**
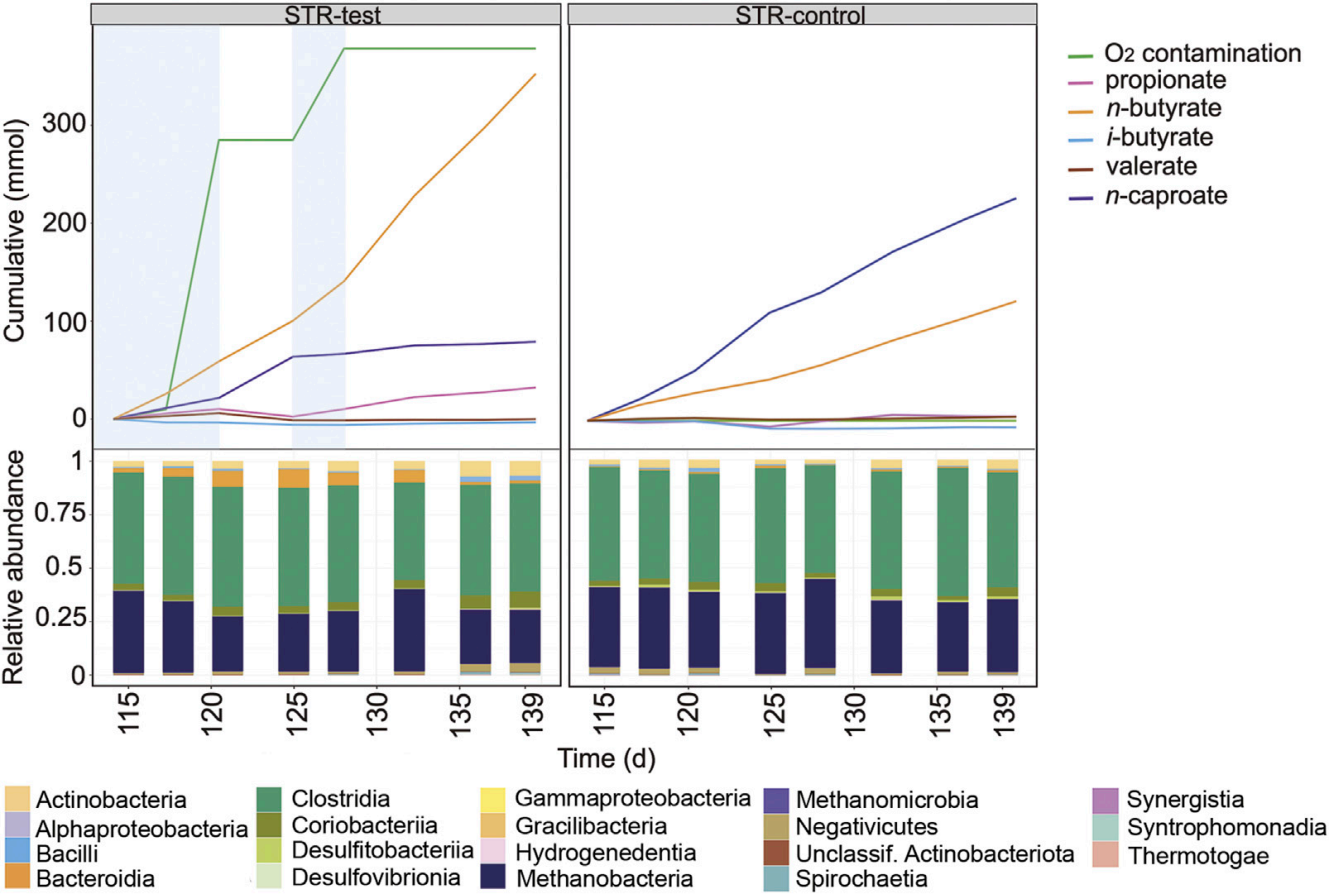
Profiles of the cumulated amounts of carboxylates and O_2_, as well as community composition at class level between days 115 and 139 for STR-test and STR-control. Blue shading indicates the O_2_ contamination period.

A second O_2_ contamination event occurred in STR-test between days 115 and 119 (Figure 3), this time for a shorter period but at double rate (471 ± 33 mL O_2_ L^−1^ d^−1^) (Supplementary Table S1). With the new contamination event in STR-test, CH_4_ and *n*-caproate production rates fell to 32% (5.33 of 16.5 mmol L^−1^ d^−1^) and 50% (1.06 of 2.12 mmol L^−1^ d^−1^) of those of the control reactor, respectively (Supplementary Table S1). This time, the O_2_ contamination coincided with an increase in *n*-butyrate formation rate from 1.51 to 2.76 mmol L^−1^ d^−1^, and instead of increasing propionate production, *n*-valerate production reached four times that of the control reactor (0.29 in relation to 0.07 mmol L^−1^ d^−1^). The short period of intense O_2_ contamination had a smaller impact on the microbial community composition and coincided with an increase in relative abundance of *Prevotella* belonging to the Bacteroidia (Supplementary Figure S5). In the last 20 days of operation of STR-test, O_2_ contamination was reduced but not completely stopped (39 ± 33 mL O_2_ L^−1^ d^−1^, Supplementary Table S1) as shown between days 125 and 128 (Figure 3). In this period, propionate production decreased to its lowest value in STR-test and *n-*butyrate production increased once more to 4.77 mmol L^−1^ d^−1^. Although CH_4_ formation increased again to the level observed during anoxic operation, *n-*caproate production could not be restored (Figure 3).

### Effect of O_2_ on the Fermentation in the Bubble Column Reactor

**FIGURE 4:**
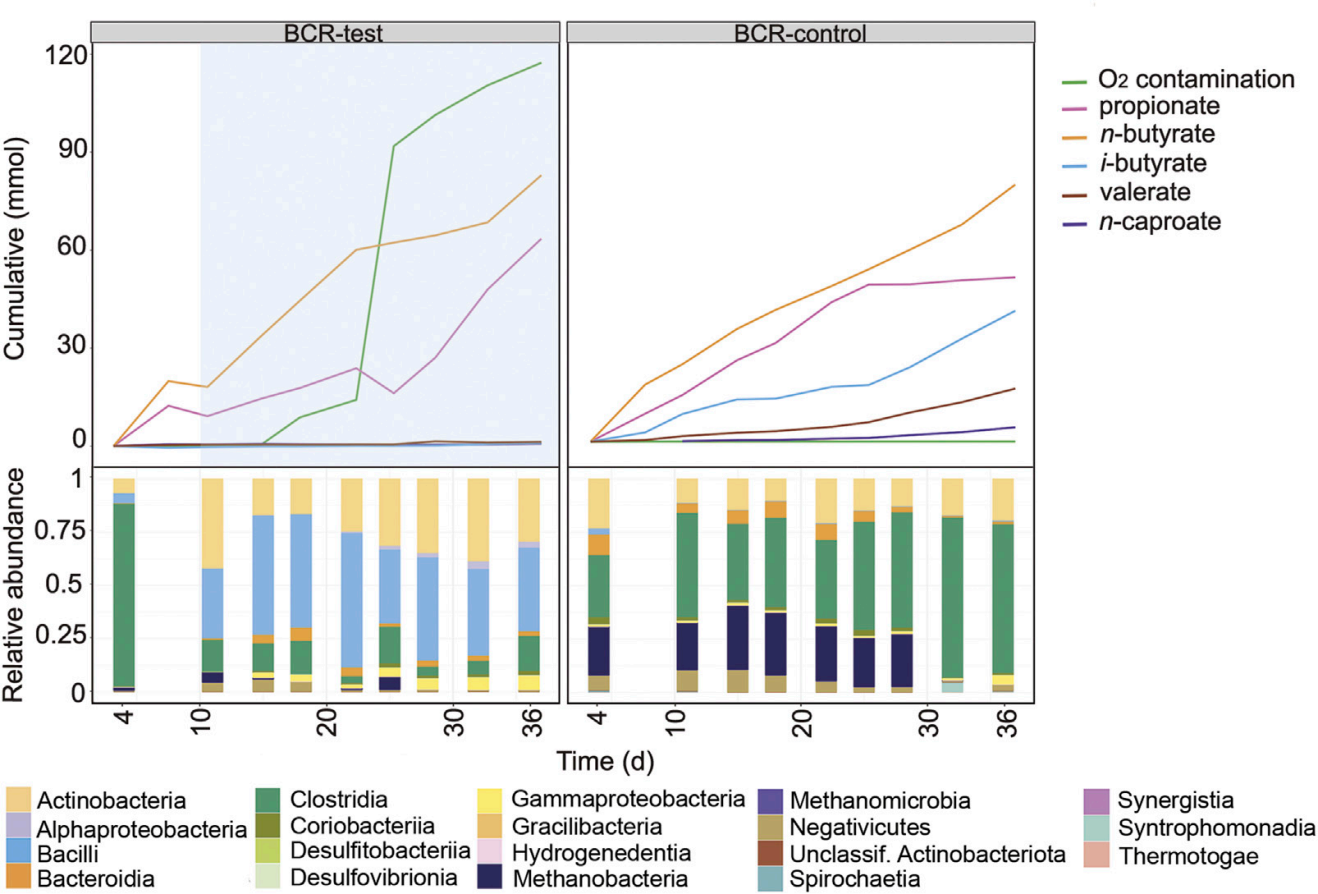
Profiles of the cumulated amounts of carboxylates and O_2_, as well as community composition at class level between days 4 and 36 for BCR-test and BCR-control. Blue shading indicates the O_2_ contamination period. Between days 11 and 28, O_2_ concentrations in the gas phase were detected up to 18% in BCR-test, hence, determination of O_2_ consumption in this time period might not be accurate.

The earlier operation phase of the BCRs is shown in Figure 4 and in Supplementary Figure S6. For this comparison period, the mixing of broths was not enough to ensure equal community compositions in BCR-test and BCR-control. The O_2_ contamination period between days 11 and 36 in BCR-test differed from the other contamination events since O_2_ concentrations up to 18% were detected in the gas phase between days 11 and 28 (Supplementary Figure S3). Considering that the assumptions for balance calculations in the reactors do not account for high O_2_ concentrations, the O_2_ contamination rate determined for this period (97 ± 28 mL O_2_ L^−1^ d^−1^) might be inaccurate. When O_2_ was detected in the system, the microbial community in the BCR-test showed a strong dominance of *Rummeliibacillus* (Supplementary Figure S6) accompanied by Actinobacteria and Gammaproteobacteria (Figure 4).

**FIGURE 5:**
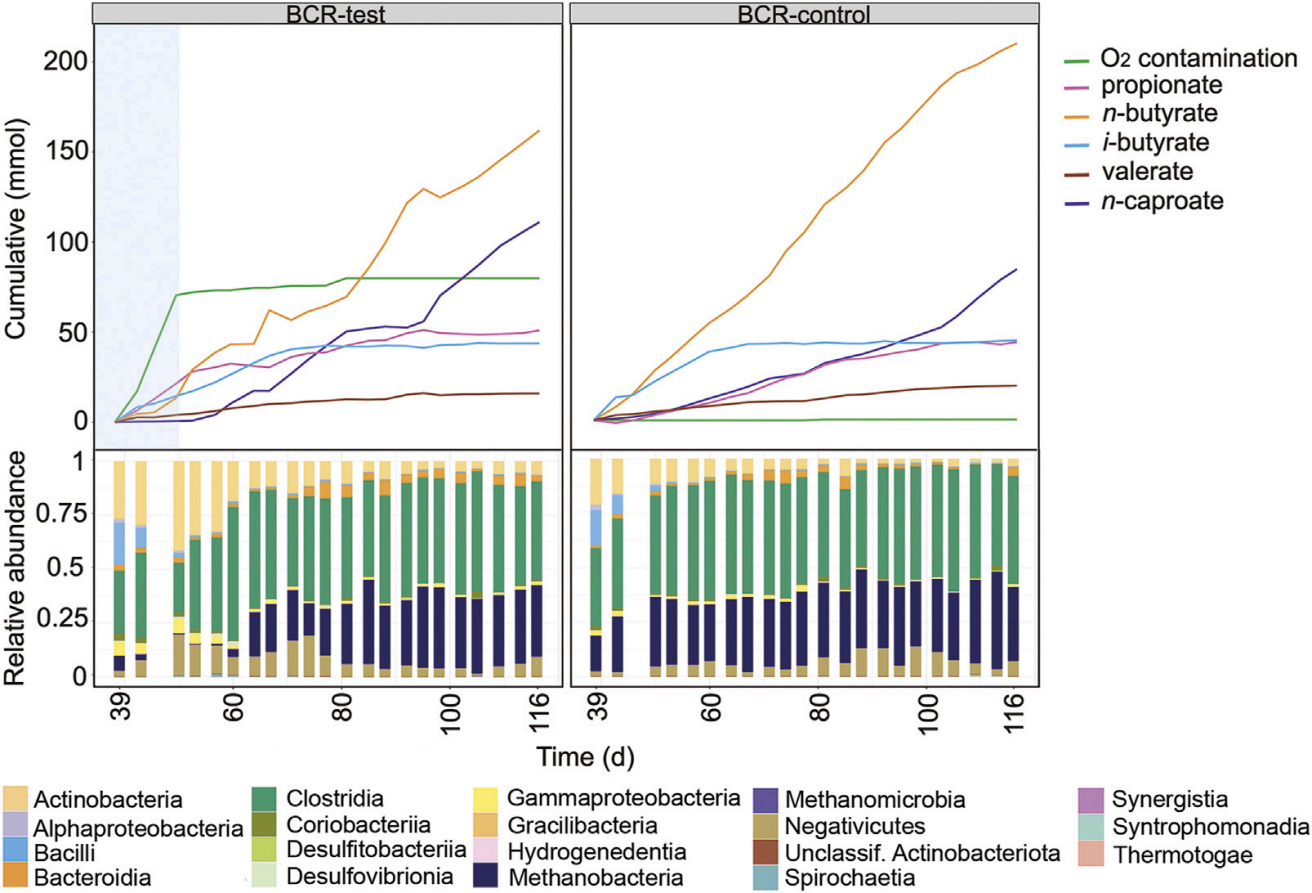
Profiles of the cumulated amounts of carboxylates and O_2_, as well as community composition at class level between days 39 and 116 for BCR-test and BCR-control. Blue shading indicates the O_2_ contamination period.

After mixing the broths of the bubble columns to start a new comparison period, BCR-test received 129 ± 28 mL O_2_ L^−1^ d^−1^ between days 39 and 50 whereas BCR-control remained completely anoxic (Figure 5). During the contamination period, BCR-test produced virtually no *n*-caproate (0.03 mmol L^−1^ d^−1^ in comparison to 0.92 mmol L^−1^ d^−1^ in control) and propionate was produced instead (1.78 mmol L^−1^ d^−1^, 2.8 times that of the control). With O_2_ contamination, BCR-test produced 46% less *n*-butyrate (1.22 of 2.25 mmol L^−1^ d^−1^) and 91% less methane (1.41 of 15.9 mmol L^−1^ d^−1^) than the control (Supplementary Table S1). After O_2_ contamination stopped in BCR-test (from day 50 on), *n*-caproate production recovered with a rate of 1.40 mmol L^−1^ d^−1^ while propionate formation decreased to 0.34 mmol L^−1^ d^−1^. *n*-Butyrate and methane production recovered partially and remained 18% (1.84 of 2.25 mmol L^−1^ d^−1^) and 25% (11.9 of 15.9 mmol L^−1^ d^−1^) lower than that of the control, respectively.

Similarly to what was seen in the STRs, Actinobacteria increased their relative abundances at the cost of Clostridia and Methanobacteria during the O_2_ contamination period in BCR-test (Figure 5). In addition, Gammaproteobacteria also became more abundant during the contamination. Visualization of the community development at genus level in Supplementary Figure S7 reveals that the main genera of Actinobacteria were the same as those found in the STRs (i.e., *Acidipropionibacterium* and *Actinomyces*). Besides the Clostridia genera that dominated in the STRs, a transient presence of *Eubacterium* was also observed in the BCRs. Gammaproteobacteria were represented by *Sutterella* and *Burkholderia* (Supplementary Figure S7).

### Correlations between Community Members and Abiotic Parameters

**FIGURE 6:**
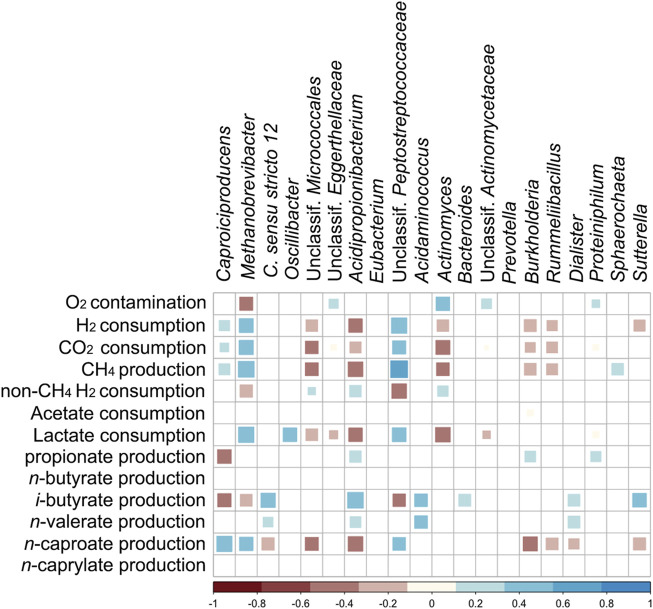
Spearman correlation matrix between the most abundant genera, O_2_ contamination rate, and production/consumption rates of chemicals (*p* < 0.01). “non-CH_4_ H_2_ consumption” stands for H_2_ consumption after discounting methane formation.

Figure 6 shows Spearman correlations between the most abundant genera, O_2_ contamination, and the formation or consumption rates of the main chemicals. In Supplementary Table S2, the correlation coefficients and their *p*-values are listed. Positive correlations of *n*-caproate formation and relative abundances of *Caproiciproducens*, unclassified *Peptostreptococcales*, and *Methanobrevibacter* were found, whereas *Clostridium sensu stricto* 12, unclassified *Micrococcales, Acidipropionibacterium*, *Burkholderia*, *Rummeliibacillus, Dialister,* and *Sutterella* correlated negatively. Propionate production correlated positively to abundances of *Acidipropionibacterium, Burkholderia*, and *Proteiniphilum*. O_2_ contamination correlated negatively with *Methanobrevibacter,* whereas positive correlations were found with abundances of unclassified *Eggerthellaceae*, unclassified *Actinomycetaceae*, *Actinomyces*, and *Proteiniphilum*. Abundances of *Clostridium sensu stricto* 12, *Acidipropionibacterium, Acidaminococcus,* and *Dialister* correlated positively with *i-*butyrate and *n-*valerate production. H_2_ consumption after discounting methane production (i.e. non-CH_4_ H_2_ consumption) correlated positively with *Acidipropionibacterium, Actinomyces*, and unclassified *Micrococcales*. *i-*Butyrate production correlated negatively with relative abundances of *Methanobrevibacter*, *Caproiciproducens*, and unclassified *Peptostreptococcales*.

Among the most abundant amplicon sequence variants (ASVs), *Clostridium sensu stricto* 12 and *Acidipropionibacterium* had ASVs that were identical to isolated species in the Silva 138 database. Within the genus *Clostridium sensu stricto* 12, ASVs were assigned to *C. luticellarii*, *C. tyrobutyricum*, or *C. ljungdahlii* (the latter was also ambiguously assigned to *C. autoethanogenum, C. ragsdalei*, and *C. coskatii*). ASVs within *Acidipropionibacterium* were assigned to *A. acidipropionici*.

## Discussion

Regardless of O_2_ contamination, the BCRs had higher concentrations of propionate, *i-*butyrate, and *n-*valerate, whereas the stirred tank design facilitated higher concentrations of *n-*butyrate, *n-*caproate, and *n-*caprylate (Table 1). The production of *n-*valerate and *i-*butyrate was not clearly related to O_2_ contamination (Figures 2, 5 and Supplementary Table S1), but its connection to *Clostridium sensu stricto* 12 (Figure 6), specifically *C. luticellarii*, was observed previously (de Smit et al., 2019; de Leeuw et al., 2020; Huang et al., 2020; Baleeiro et al., 2021a). *C. luticellarii* was shown to be important for *n-*valerate production from propionate (de Smit et al., 2019) and could therefore play an important role in the production of odd-chain MCC in chain elongation reactors. *n-*Valerate and *i-*butyrate production also correlated with other genera that are not commonly known for the production of these compounds (Figure 6). Higher relative abundances of *Caproiciproducens* not only correlated significantly with *n*-caproate production (Figure 6) but were visually related to higher concentrations of *n*-caproate (Supplementary Figures S4, S7). The correlation of *n*-caproate production with the abundance of *Caproiciproducens* is not surprising, since this genus has been commonly found in other MCC-producing communities (Duber et al., 2020; Joshi et al., 2021).

Micro-aerobic conditions favored the classes Actinobacteria, Gammaproteobacteria, Bacilli, and Coriobacteriia over Clostridia and Methanobacteria. Notably, a similar pattern is known for gut microbiota, where Actinobacteria and Alpha- or Gammaproteobacteria have been observed to dominate over Clostridia in regions of the gut more exposed to O_2_ (Friedman et al., 2018). One explanation is that the evolutionary younger taxa of aerotolerant Actinobacteria (e.g., propionibacteria) and Proteobacteria (Martin and Sousa, 2015) are generally better equipped with enzymes that mitigate the toxicity of ROS (e.g., catalases, H_2_O_2_ reductases, and superoxide dismutases) than typical strict anaerobes such as Clostridia (Kato et al., 1997; Johnson and Hug, 2019).

### Partial Acclimatization of Methanogens to Ethylene

Hydrogenotrophic methanogenesis was likely the main pathway for CH_4_ production. This is supported by the results of previous experiments where *Methanobrevibacter* was one of the predominating community members under similar conditions with H_2_/CO_2_ (Baleeiro et al., 2021a). Notably, methanogens partially overcame the inhibition by ethylene. The acclimatization occurred after 42 days of successful inhibition reported by Baleeiro et al. (2021b). To the best of our knowledge, such acclimatization has not been reported before. With ethylene, CH_4_ production was relatively strong (up to 19.5 mmol L^−1^ d^−1^) but still about one third lower than the rates observed in the absence of the inhibitor in a similar gas recirculation system (up to 32.8 mmol L^−1^ d^−1^) (Baleeiro et al., 2021b). We hypothesize that *Methanobrevibacter*, the main methanogenic genus found in our reactors, may have acclimatized to ethylene by expressing [Fe]-hydrogenases. For hydrogenotrophic methanogens, [Fe]-hydrogenases have less favorable kinetics than [NiFe]-hydrogenases. Still, some methanogens, including Methanobacteria, can express [Fe]-hydrogenases to grow under nickel-limiting conditions (Thauer et al., 2010). As postulated by Baleeiro et al. (2021b), ethylene might not exert an inhibitory effect on nickel-free hydrogenases as it does on [NiFe]-hydrogenases of methanogens. Considering that [Fe]-hydrogenases are not inhibited by O_2_ (Thauer et al., 2010; Stiebritz and Reiher, 2012), micro-aerobic conditions and broth mixing between reactors could have caused further selection of methanogens expressing [Fe]-hydrogenases even if nickel was not limiting. Further studies using transcriptome or proteome analyses of pure methanogenic cultures are needed to test this hypothesis.

### Steering the Fermentation with Small O_2_ Contamination

When the O_2_ concentration in the gas phase increased up to 18% between days 11 and 28, the aerobic genus *Rummeliibacillus* (Vaishampayan et al., 2009; Her and Kim, 2013) flourished in the bubble column reactor and the concentrations of *n*-butyrate and propionate decreased (Supplementary Figure S6). With an O_2_ concentration below the detection limit by day 28 (Supplementary Figure S3), Actinobacteria abundance increased and propionate production reached its highest rates (Supplementary Table S1).

Methanogenesis and *n*-caproate production were strongly inhibited by O_2_ intrusion. After the contamination stopped, CH_4_ production recovered on every occasion but *n*-caproate production did not (Supplementary Table S1), indicating that the O_2_ contamination had particularly strong detrimental effects on C4-to-C6 chain elongation. In the period between days 119 and 139 in STR-test, *n*-caproate production did not increase after O_2_ contamination rate changed from 474 ± 33 mL O_2_ L^−1^ d^−1^ to 39 ± 33 mL O_2_ L^−1^ d^−1^, instead, the highest rates of *n-*butyrate production and acetate consumption were achieved. This was observed even with high relative abundances of *Caproiciproducens* in the community (Supplementary Figure S5).

Relatively low O_2_ contamination rates were found to favor propionate formation in lactate-based fermentation. However, the relationship between O_2_ and propionate accumulation was not as straightforward as the inhibitory effect of O_2_ on chain elongation and methanogenesis. One possible reason is that propionate is not only a product of lactate fermentation, but also a substrate for *n*-valerate production by chain-elongating bacteria (Angenent et al., 2016). This could explain what was observed in the micro-aerobic period between days 115 and 119 in the STR-test, when propionate production did not increase but *n-*valerate production was relatively high (Supplementary Table S1).

### Key Players that can Profit from O_2_ Intrusion

Among the microorganisms enriched in the O_2_ contamination periods, *Acidipropionibacterium* correlated to propionate production (*p* < 0.01). Other Actinobacteria enriched during O_2_ contamination did not correlate with propionate production and may have diverted electrons from lactate to products other than propionate, which could be another reason why STR-test showed lower propionate production rates (Supplementary Table S1). *Actinomyces,* a facultative anaerobe (Rao et al., 2012), was particularly enriched in STR-test during an O_2_ contamination period (Supplementary Figure S4). *Actinomyces* can grow anaerobically or aerobically on lactate and produces acetate, formate, and CO_2_ during fermentative growth (Takahashi et al., 1994; Takahashi and Yamada, 1999; Rao et al., 2012). In agreement with the reported aerobic growth of *Actinomyces naeslundii* on lactate (Takahashi and Yamada, 1999), *Actinomyces* was likely not responsible for propionate production in the STR. The Coriobacteriia (*Eggerthellaceae*) observed in O_2_ contamination periods belong to a family of strict anaerobes that are not reported to produce propionate (Gupta et al., 2013). *Proteiniphilum* (as well as *Dialister*) are genera with microaerophilic species that produce propionate, although it is not clear if from lactate (Tomazetto et al., 2018; Sakamoto et al., 2020). In our study, the abundance of *Proteiniphilum* correlated to O_2_ contamination and to propionate formation whereas no significant correlation was found between *Dialister*, propionate, and O_2_ contamination (Figure 6).

Fermentation of lactate by propionate producing bacteria commonly leads to a 2:1:1 stoichiometry of propionate to acetate to CO_2_ (Eq. 2). Gammaproteobacteria and Actinobacteria species that produce propionate are known to use methylmalonyl-CoA pathways rather than the acrylate pathway (Seeliger et al., 2002; Gonzalez-Garcia et al., 2017). In particular, *Acidipropionibacterium* spp. are among the most efficient propionate producers thanks to the highest energy efficiency of their methylmalonyl-CoA pathway (also known as Wood-Werkman cycle, a succinate pathway involving methylmalonyl-CoA:pyruvate transcarboxylase) (Scholz and Kilian, 2016; Gonzalez-Garcia et al., 2017).

$$3\;CH_{3}CHOHCOO^{-}\to 2\;CH_{3}CH_{2}COO^{-}+CH_{3}COO^{-}+CO_{2}+H_{2}O$$ (2)

Even though it can express O_2_-sensitive enzymes for fermentative growth similar to those in *Clostridium*, *Acidipropionibacterium* also has aerotolerant enzymes with similar functions (Piwowarek et al., 2018; McCubbin et al., 2020). Members of this genus are not only more tolerant to O_2_ than clostridia, they have also been found to increase propionate and energy yields when exposed to O_2_ (McCubbin et al., 2020).

It should be taken into account that propionibacteria, unlike many clostridia, do not form endospores (Gonzalez-Garcia et al., 2017). Hence, if exploration of propionate production is desired, shock treatments of the inoculum (e.g., with pH or heat), common techniques for starting non-methanogenic anaerobic bioprocesses (Baleeiro et al., 2019), should be avoided.

The phenomenon that lactate is diverted to propionate in chain elongation reactors under micro-aerobic conditions may have been overlooked in former studies. In one notable case, Kucek et al. (2016) observed the competitive production of propionate in a lactate-based chain elongation reactor. Although the possibility of O_2_ contamination was not discussed, the study detected high abundances of *Acinetobacter*, strictly aerobic Gammaproteobacteria (Smet et al., 2014) commonly found in micro-aerated reactors (Krayzelova et al., 2015). To explain the propionate production observed in certain time periods, Kucek et al. (2016) suggested the residual concentration of lactate in the reactor to be a determining factor. Although not discussed in the study, O_2_ presence could have played a role in propionate production.

### Possible Roles of H_2_ during O_2_ Contamination

A common way for the reduction of O_2_ in the presence of H_2_ is shown in Eq. 3 and is realized even by obligate anaerobes such as methanogens (Thauer et al., 2010), Negativicutes (Boga et al., 2007), and sulfate reducers (Dannenberg et al., 1992; Chen et al., 1993).

$$2H_{2}+O_{2}\to 2H_{2}O$$ (3)

H_2_ consumption that was not attributed to methane formation was particularly high during O_2_ contamination periods. In STR-test, it ranged from 3.0 to 3.4 fold molar O_2_ consumption, while in BCR-test it ranged from 1.1 to 3.6 fold. Considering the 2:1 ratio of H_2_ to O_2_ during H_2_ oxidation (Eq. 3), H_2_ consumption not linked to methane formation in this study may have been related to other reactions during O_2_ intrusion. Interestingly, similar consumption ratios of H_2_ to O_2_ (between 3.2 and 3.4) have been observed in communities dominated by hydrogen-oxidizing bacteria during autotrophic growth (Matassa et al., 2016). Nevertheless, we did not observe the presence of *Sulfuricurvum* (the genus enriched by Matassa et al. (2016)) and *Burkholderia*, a possible aerobic autotroph (Takors et al., 2018) found in our study, did not correlate positively with H_2_ consumption nor with O_2_ contamination. Besides, if aerobic hydrogen-oxidizing bacteria played a major role in the micro-aerobic reactors in our study, signs of biomass formation and carbon source (e.g., CO_2_) consumption should have accompanied H_2_ oxidation with O_2_. However, no clear relation between O_2_ contamination, biomass formation, and CO_2_ consumption rates was found.

Communities enriched with propionate-producing bacteria in anaerobic reactors, such as *Acidipropionibacterium* spp., often correlate with high H_2_ consumption or low H_2_ production (Cabrol et al., 2017). In the presence of exogenous H_2_, some propionate producers such as *Propionispira arboris* are able to perform homopropionate fermentation of lactate (Eq. 4) producing neither CO_2_ nor acetate (Thompson et al., 1984).

$$CH_{3}CHOHCOO^{-}+H_{2}\to CH_{3}CH_{2}COO^{-}+H_{2}O$$ (4)

We did not observe *Propionispira* spp. in our reactors and its closest relative found in our system (*Dialister*) is only related at the order level (*Veillonellales-Selenomonadales*). Besides, Eq.4 alone cannot explain the high H_2_ consumption during most of the micro-aerobic periods in this study. H_2_ consumption not linked to methane was much higher than propionate formation. In fact, the period between days 39 and 50 of BCR-test had the lowest H_2_ to O_2_ consumption ratio and was the period with the highest propionate productivity (Supplementary Table S1). Lastly, it is not clear if the correlation found between abundance of *Acidipropionibacterium* and H_2_ consumption not linked to methane (Figure 6) is a direct one. H_2_ consumption by isolated members of *Propionibacterium* is not observed during fermentative growth (Seeliger et al., 2002).

Homoacetogens consume H_2_ and CO_2_ (Eq. 5) and, among them, at least *C. ljungdahlii* was shown to have some resistance against O_2_ exposure (Whitham et al., 2015). Here, similar clostridia were detected in the reactors and *Clostridium sensu stricto* 12 was still present during some O_2_ contamination events (Supplementary Figures S3, S7). Therefore, homoacetogenic activity could be considered to explain H_2_ consumption during O_2_ contamination. Nevertheless, no further evidence for this hypothesis was found. H_2_ consumption was not accompanied by net CO_2_ consumption and the net acetate production was unfavorable because acetate was fed in excess with the growth medium (12 g L^−1^ acetate) to favor chain elongation as an acetate-consuming reaction.

$$4H_{2}+2CO_{2}\to CH_{3}COO^{-}+H^{+}+2H_{2}O$$ (5)

Another way that H_2_ presence might have influenced the micro-aerated community is by amplifying the effects of O_2_ contamination. The activation of O_2_ into ROS by hydrogenases and reduced electron carriers might be accentuated by H_2_ recirculation (Misra and Fridovich, 1971; Krab et al., 1982). Since we did not have a control reactor for the presence of H_2_, we could not test this hypothesis.

## Conclusion

Even small O_2_ contaminations were very detrimental to *n*-caproate and methane formation, but favored propionate formation. The relation of O_2_ contamination and propionate formation was not straightforward: reactors with micro-aerobic conditions produced more propionate overall, but propionate production cycles were not always synchronous to O_2_ contamination. Besides, the negative effects of O_2_ on methane formation could be reversed in all cases whereas chain elongation could not always be resumed when O_2_ contamination stopped. These patterns were observed in both stirred tank and in bubble column reactors, with the bubble column process being more sensitive to O_2_ contamination.

It is unclear whether the effects of O_2_ reported in this study could be reproduced without the recirculation of H_2_. It is possible that the H_2_ recirculation amplified the effects of O_2_ toxicity, since presence of H_2_ can favor ROS formation by hydrogenases. Considering that H_2_ consumption was particularly high during micro-aeration, H_2_ may have acted as an important energy source for aerotolerant and aerobic microorganisms. Controlled O_2_ contamination studies with co-cultures or mixed communities of lower complexity can shed light on how impactful H_2_ recirculation during micro-aeration is.

Aerotolerant fermenting bacteria such as *Acidipropionibacterium* spp. are efficient propionate producers that could be regarded as welcomed guests rather than competitors. Here, they were the main candidates responsible for propionate production although their correlation with O_2_ contamination was unclear. Instead, *Actinomyces* spp. (Actinobacteria that did not produce propionate) profited most from the micro-aerobic environment. Future experiments could help clarify if stable propionate-producing communities can be selected by micro-aeration. If micro-aeration facilitates propionate accumulation, a sequential anaerobic step can be used for chain elongation with high selectivity to odd-numbered MCC. Studies with defined cultures aiming to understand what is behind the recovery of chain elongation activity after micro-aerobic periods are also recommended.

## Data Availability

The datasets generated for this study can be found in the European Nucleotide Archive (ENA) under accession number PRJEB44209 (http://www.ebi.ac.uk/ena/data/view/PRJEB44209).
