# Supplementary material for: Effect of Oxygen Contamination on Propionate and Caproate Formation in Anaerobic Fermentation
Source: Front Bioeng Biotechnol. 2021 Sep 10;9:725443. doi: 10.3389/fbioe.2021.725443 (PMC8460912; doi:10.3389/fbioe.2021.725443)
Supplement: Supplementary file 1 [file DataSheet1.pdf]

Supplementary Material

1 Supplementary Figures and Tables

1.1 Supplementary Figures

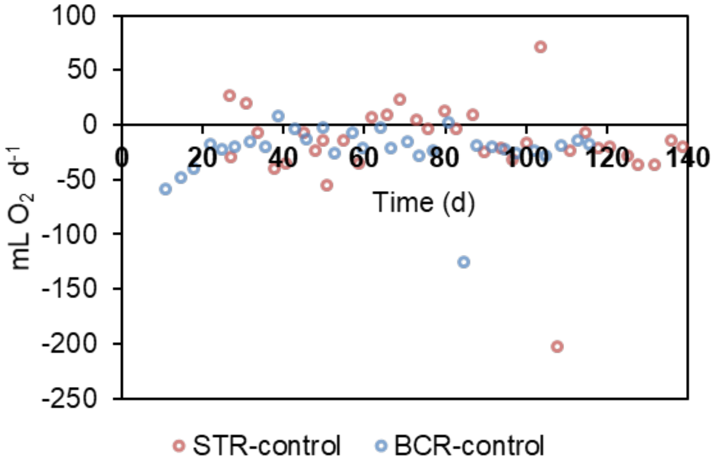

Supplementary Figure 1. Distribution of estimated oxygen contamination rates in the reactors STR-test and BCR-test. These reactors remained anoxic. Thus, the distribution was used to determine the standard error of the procedure for quantifying oxygen contamination rates in STR-test and BCR-test.

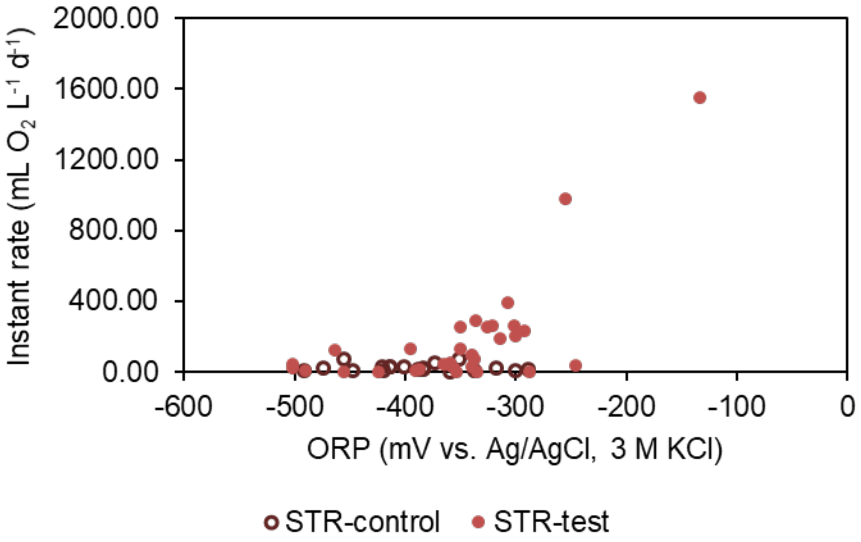

Supplementary Figure 2. Instant O_2 contamination rate versus oxidation-reduction potential (ORP) measurement. No clear relation between O_2 contamination rates and ORP measurements was found at lower contamination rates.

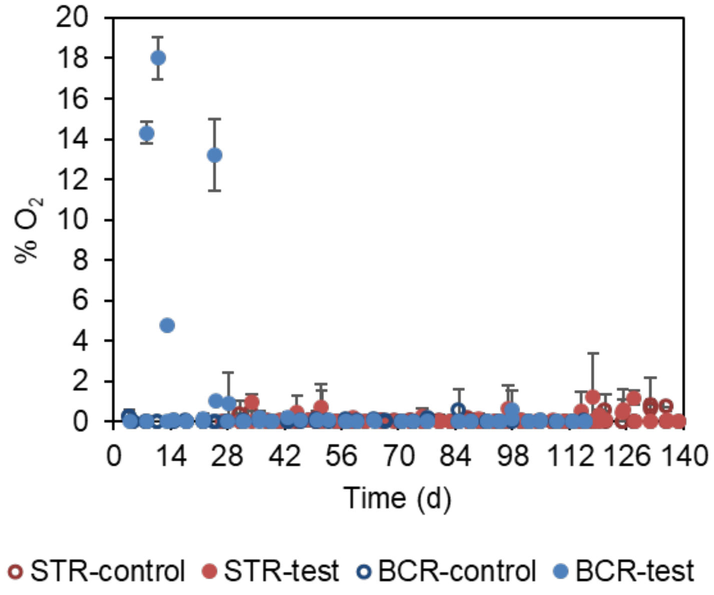

Supplementary Figure 3. O_2 concentrations in the recirculating gas.

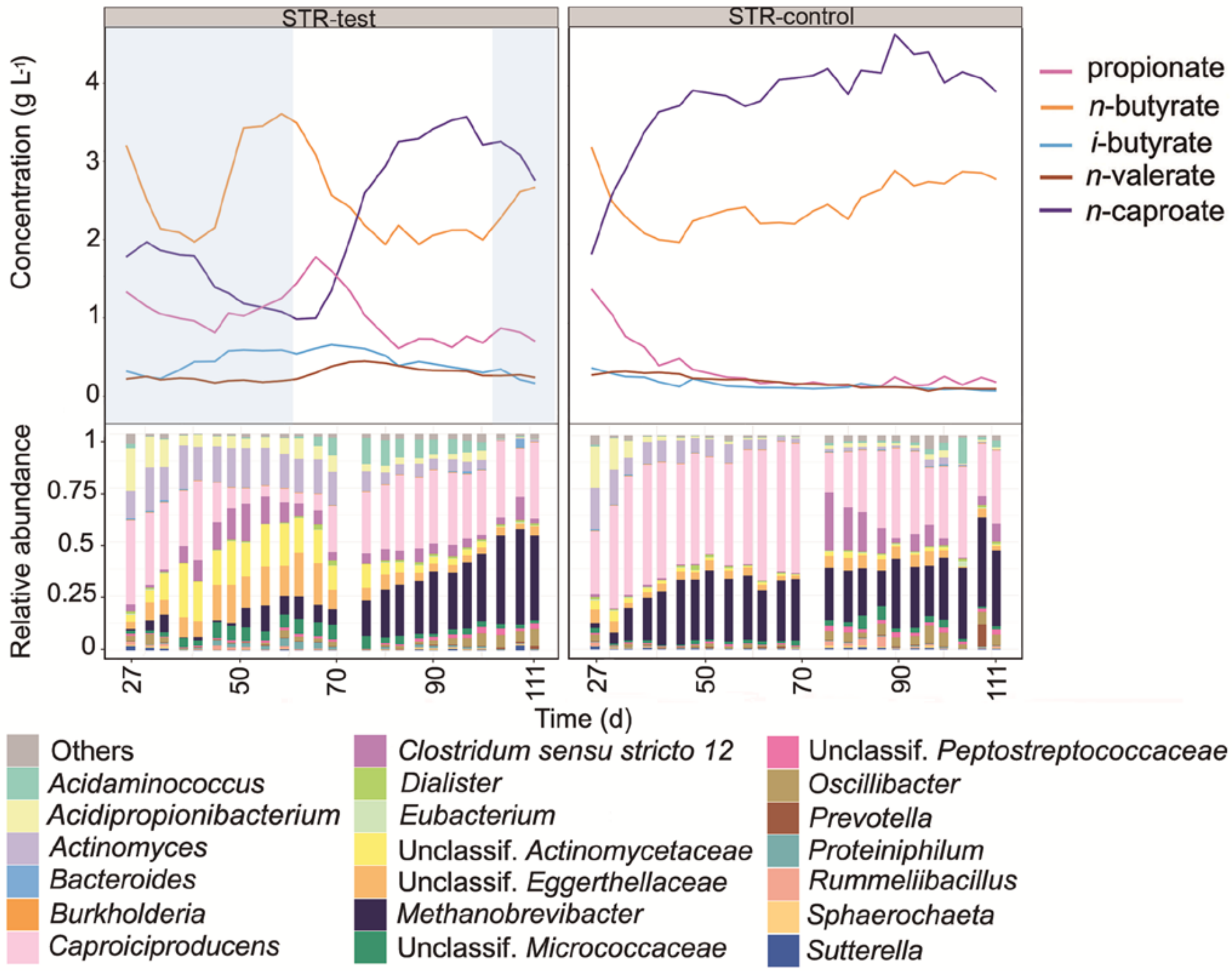

Supplementary Figure 4. Concentrations of the main carboxylates produced and community composition at genus level between days 27 and 111 for STR-test and STR-control. Blue shading indicates the O_2 contamination period.

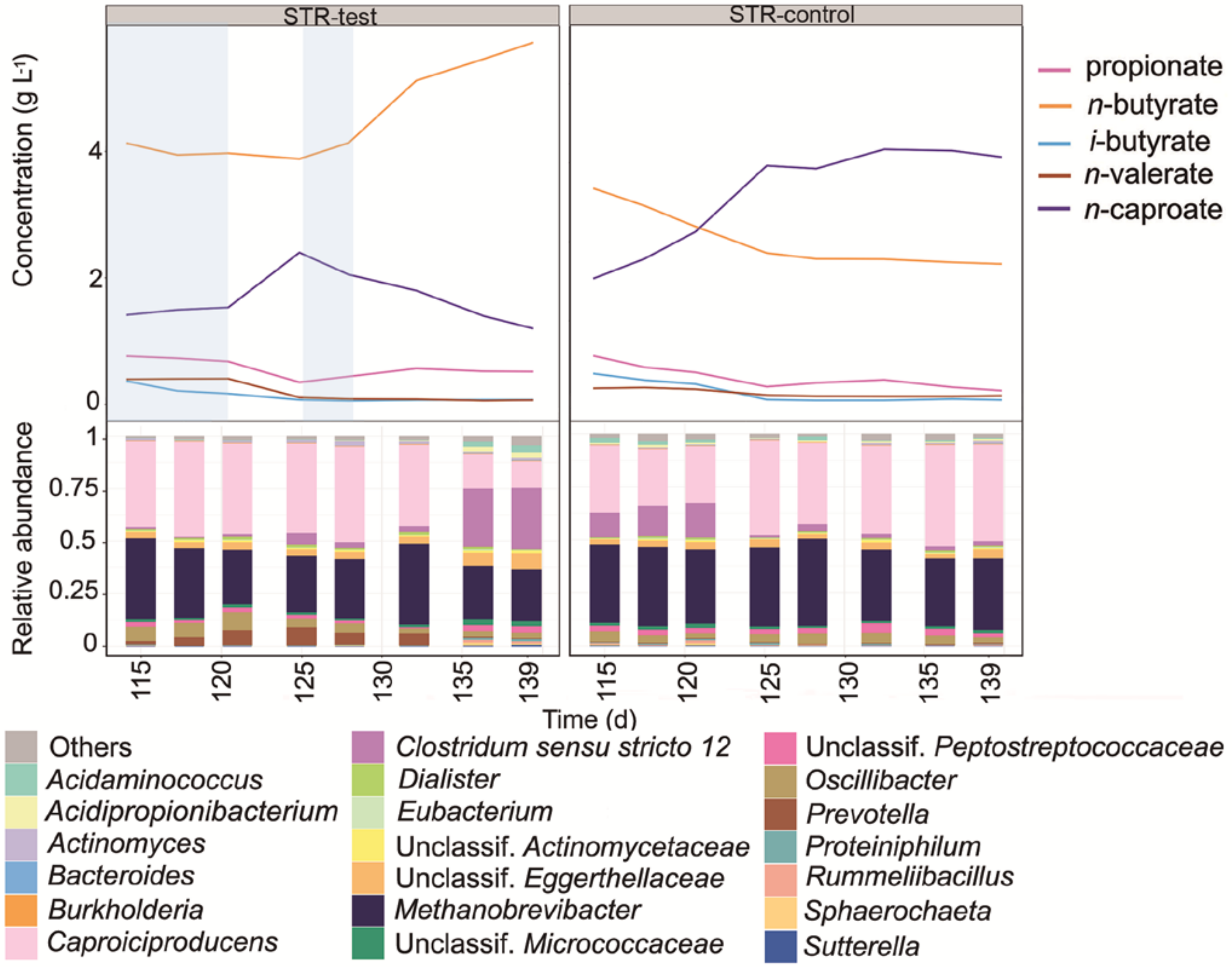

Supplementary Figure 5. Concentrations of the main carboxylates produced and community composition at genus level between days 115 and 139 for STR-test and STR-control. Blue shading indicates the O_2 contamination period.

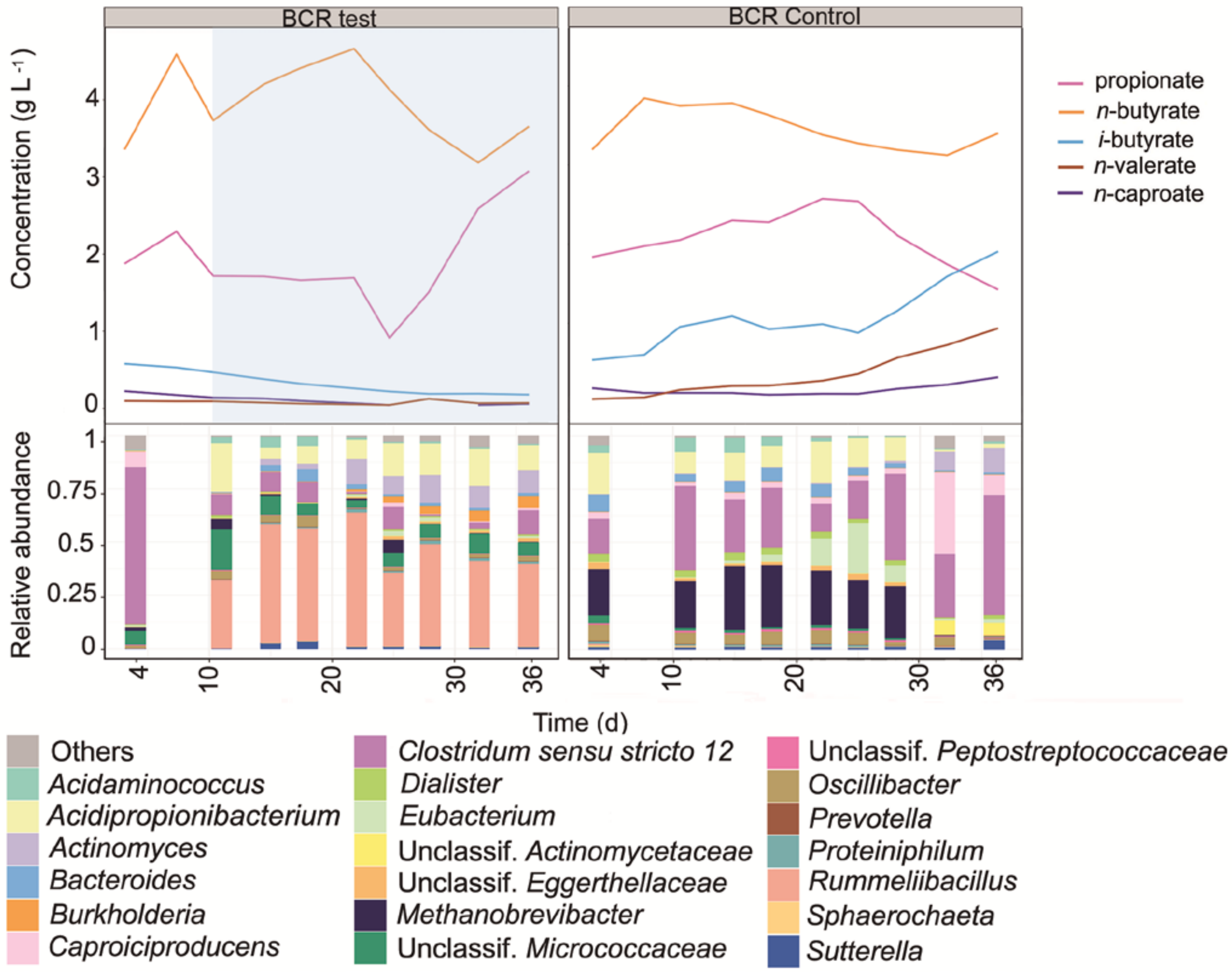

Supplementary Figure 6. Concentrations of the main carboxylates produced and community composition at genus level between days 4 and 36 for BCR-test and BCR-control. Blue shading indicates the O_2 contamination period.

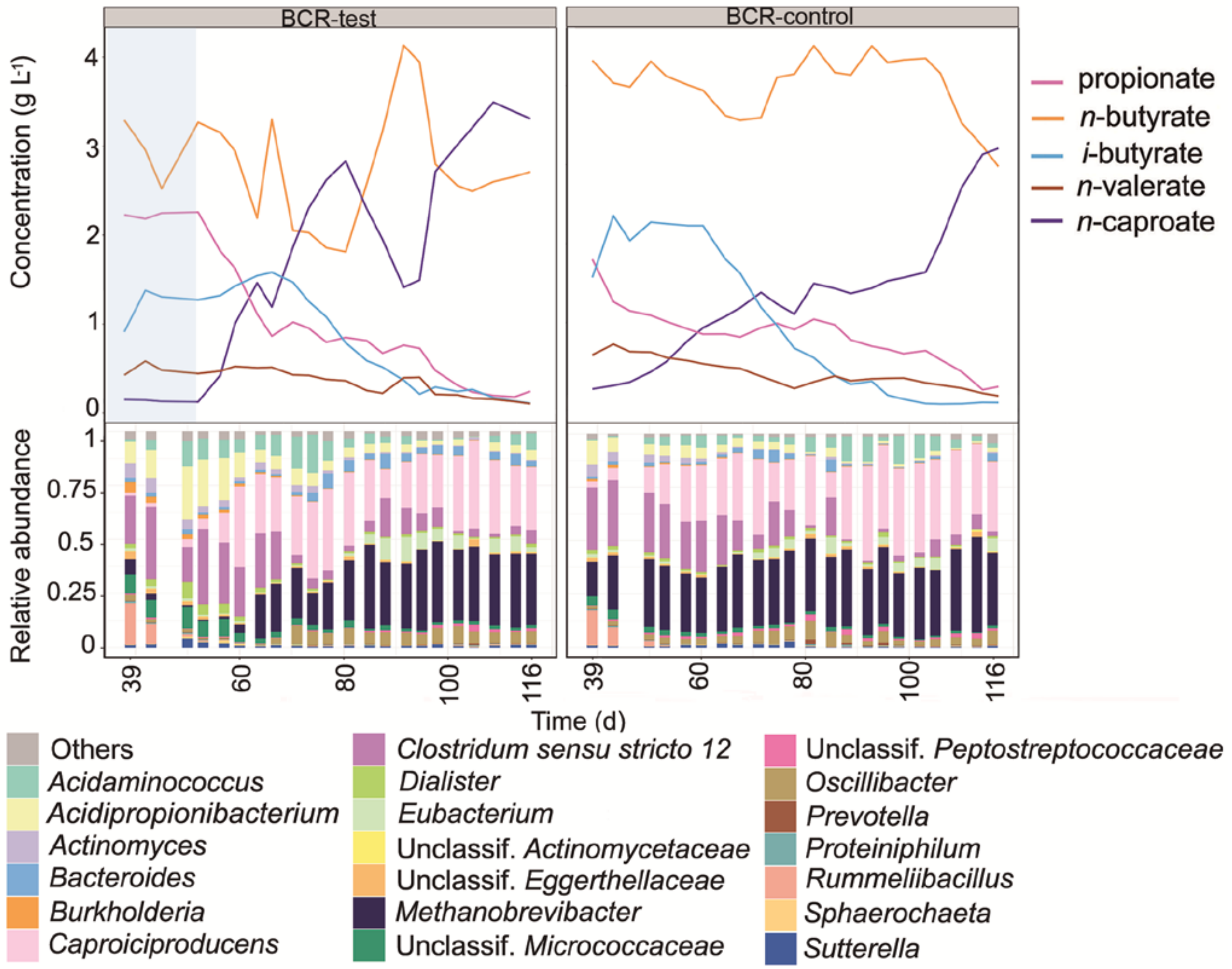

Supplementary Figure 7. Concentrations of the main carboxylates produced and community composition at genus level between days 39 and 116 for BCR-test and BCR-control. Blue shading indicates the O_2 contamination period.

1.2 Supplementary Table

Supplementary Table 1. Average specific rates for each reactor and period. Values in bold correspond to periods with the highest O_2 contamination. Positive values are production rates and negative values are consumption rates. Formate, ethanol, *n*-butanol, *i*-caproate, and *n*-heptanoate were also monitored but had negligible rates. "non-CH_4 H_2" stands for hydrogen after discounting methane formation. Shorter O_2 contamination periods occurred in STR-test during 104 - 111 d and 125 - 128 d but are not shown separately. *During this period, O_2 concentrations of up to 18% were detected in the gas phase, since O_2 concentrations below detection level are assumed in the estimation of the O_2 contamination rate, the value shown may not be accurate.

Reactor	Period (days)	Specific O_2 contamination rate (mL L^{-1} d^{-1})	Average specific rate (mmol L^{-1} d^{-1})													
			O_2	H_2	CO_2	CH_4	non-CH_4 H_2	Biomass	Acetate	Lactate	Propionate	*n*-Butyrate	*i*-Butyrate	*n*-Valerate	*n*-Caproate	*n*-Caprylate
STR-test	**27 – 59**	**220 ± 33**	**-8.86**	**-50.5**	**-0.2**	**6.05**	**-26.3**	**1.48**	**-1.80**	**-7.52**	**0.76**	**1.78**	**0.36**	**0.10**	**0.56**	**0.02**
	59 - 111	21 ± 33	-0.87	-71.2	-10.2	19.5	7.07	1.72	-2.18	-8.92	0.67	1.51	0.23	0.21	1.78	0.02
	**115 - 119**	**474 ± 33**	**-19.1**	**-86.1**	**-3.4**	**5.33**	**-64.7**	**2.03**	**-2.65**	**-9.88**	**0.61**	**2.76**	**-0.19**	**0.29**	**1.06**	**-0.02**
	119 - 139	39 ± 33	-1.56	-76.8	-12.7	18.7	-1.95	1.74	-4.38	-9.85	0.36	4.77	-0.01	-0.07	0.97	0.11
STR-control	27 - 139	7 ± 33	-0.21	-62.3	-10.7	16.5	3.73	1.37	-2.91	-8.63	0.13	1.71	0.06	0.07	2.12	0.11
BCR-test	4 – 11	0 ± 28	-0.01	-31.2	-5.51	8.73	3.72	1.30	0.42	-7.58	1.10	2.18	-0.04	0.06	0.06	0.00
	**11 – 36 ***	**97 ± 28 ***	**-3.9 ***	**-15.9**	**1.2**	**0.42**	**-14.2**	**1.21**	**-1.39**	**-6.39**	**1.37**	**1.98**	**0.27**	**0.13**	**0.04**	**0.00**
	**39 – 50**	**129 ± 28**	**-5.2**	**-11.5**	**9.3**	**1.41**	**-5.86**	**0.54**	**-1.63**	**-7.13**	**1.78**	**1.22**	**1.08**	**0.29**	**0.03**	**0.00**
	50 - 116	3 ± 28	-0.12	-47.2	-8.5	11.9	0.27	1.24	-2.32	-8.02	0.34	1.84	0.37	0.15	1.40	0.00
BCR-control	4 – 116	0 ± 28	-0.01	-63.9	-12.71	15.9	-0.50	1.13	-1.98	-7.54	0.75	2.25	0.60	0.25	0.66	0.00

Supplementary Table 2. Spearman correlation coefficients and their p-values (in parentheses).

	Caproici-producens	*Methano-brevibacter*	*C. sensu stricto 12*	*Oscillibacter*	*Unclassif. Micro-coccales*	*Unclassif. Egger-thellaceae*	*Acidipropioni-bacterium*	*Eubacterium*	*Unclassif. Peptostrepto-coccaceae*	*Acidamino-coccus*	*Actinomyces*
O_2 contamination	-0.071 (0.61)	-0.368 (0.002)	-0.121 (0.675)	-0.237 (0.488)	0.315 (0.032)	0.183 (9.63E-6)	0.115 (0.882)	-0.246 (0.145)	-0.215 (0.703)	-0.188 (0.021)	0.389 (1.45E-5)
H_2 consumption	0.233 (1.95E-3)	0.411 (1.36E-7)	-0.014 (0.73)	0.152 (0.371)	-0.29 (4.73E-4)	0.14 (0.582)	-0.392 (5.10E-5)	-0.082 (0.456)	0.504 (3.88-08)	0.108 (0.902)	-0.291 (2.15E-3)
CO_2 consumption	0.17 (1.55E-3)	0.434 (5.7E-11)	0.04 (0.875)	0.218 (0.086)	-0.339 (2.02E-6)	-0.068 (0.001)	-0.266 (4.33E-3)	0.101 (0.476)	0.369 (1.05E-4)	0.258 (0.169)	-0.423 (1.75E-9)
CH_4 production	0.263 (6.50E-4)	0.539 (4.8E-12)	-0.038 (0.585)	0.212 (0.241)	-0.353 (2.14E-8)	0.12 (0.762)	-0.461 (3.01E-7)	-0.021 (0.905)	0.601 (8.5E-11)	0.189 (0.398)	-0.35 (1.77E-5)
non-CH_4 H_2 consumption	-0.134 (0.163)	-0.33 (7.34E-5)	0.047 (0.63)	-0.175 (0.438)	0.119 (2.72E-6)	-0.082 (0.13)	0.287 (3.66E-3)	0.028 (0.294)	-0.453 (3.18E-3)	-0.211 (0.063)	0.237 (2.43E-3)
Acetate consumption	0.072 (0.2)	0.202 (0.037)	-0.053 (0.651)	0.116 (0.11)	-0.152 (0.865)	0.001 (0.798)	-0.228 (0.067)	0.062 (0.813)	0.153 (0.159)	-0.104 (0.725)	-0.137 (0.076)
Lactate consumption	0.18 (0.107)	0.501 (1.20E-6)	-0.018 (0.341)	0.4 (0)	-0.302 (5.42E-3)	-0.151 (6.9E-3)	-0.395 (1.80E-4)	0.109 (0.21)	0.408 (5.08E-5)	0.167 (0.17)	-0.454 (2.30E-7)
Propionate production	-0.404 (6.71E-7)	-0.107 (0.019)	0.271 (0.015)	0.074 (0.721)	0.162 (0.159)	-0.15 (0.604)	0.286 (9.34E-5)	0.218 (0.162)	-0.069 (0.077)	0.115 (0.539)	0.055 (0.073)
n-butyrate production	-0.04 (0.471)	0.149 (0.357)	0.112 (0.234)	0.192 (0.257)	-0.077 (0.509)	-0.2 (0.413)	-0.172 (0.57)	0.17 (0.504)	0.215 (0.078)	0.059 (0.796)	-0.179 (0.084)
i-butyrate production	-0.375 (4.63E-3)	-0.304 (3.84E-3)	0.436 (1.45E-8)	-0.099 (0.591)	0.384 (0.0172)	-0.133 (0.189)	0.532 (8.72E-5)	0.161 (0.915)	-0.334 (3.66E-4)	0.343 (0.001)	0.161 (0.956)
n-valerate production	-0.046 (0.212)	-0.084 (0.059)	0.21 (6.42E-5)	0.053 (0.616)	-0.001 (0.285)	-0.211 (0.138)	0.254 (4.86E-3)	0.2 (0.717)	-0.186 (0.057)	0.344 (0.001)	-0.006 (0.729)
n-caproate production	0.498 (2.01E-8)	0.391 (1.38E-5)	-0.327 (1.10E-4)	0.101 (0.474)	-0.366 (1.79E-5)	0.25 (0.848)	-0.461 (1.06E-6)	-0.179 (0.573)	0.343 (1.88E-3)	-0.08 (0.439)	-0.151 (0.069)
n-caprylate production	0.162 (0.061)	0.064 (0.769)	-0.181 (0.205)	-0.027 (0.568)	-0.109 (0.299)	0.155 (0.755)	-0.141 (0.936)	-0.137 (0.346)	0.076 (0.997)	-0.19 (0.101)	0.038 (0.477)

Supplementary Table 2 (continued).

	Bacteroides	*Unclassif. Actino-mycetaceae*	*Prevotella*	*Burkholderia*	*Rummeli-bacillus*	*Dialister*	*Proteini-philum*	*Sphaero-chaeta*	*Sutterella*
O_2 contamination	-0.201 (0.075)	0.215 (2.23E-7)	0.016 (0.131)	0.099 (0.94)	0.09 (0.955)	-0.17 (0.422)	0.131 (0.006)	-0.104 (0.245)	-0.147 (0.166)
H_2 consumption	-0.153 (0.035)	0.195 (0.604)	0.177 (0.329)	-0.317 (4.27E-6)	-0.231 (1.53E-7)	-0.015 (0.326)	0.024 (0.253)	0.21 (0.018)	-0.268 (5.07E-4)
CO_2 consumption	0.079 (0.675)	-0.038 (2.07E-5)	0.068 (0.676)	-0.215 (2.17E-5)	-0.254 (9.10E-6)	0.105 (0.724)	-0.078 (0)	0.185 (0.016)	-0.075 (0.156)
CH_4 production	-0.121 (0.065)	0.157 (0.078)	0.213 (0.732)	-0.303 (2.01E-5)	-0.266 (5.96E-8)	-0.028 (0.297)	0.069 (0.311)	0.294 (0.002)	-0.191 (0.010)
non-CH_4 H_2 consumption	0.066 (0.968)	-0.115 (0.011)	-0.089 (0.327)	0.223 (0.548)	0.069 (0.099)	0.133 (0.694)	-0.152 (0.938)	-0.292 (0.052)	0.059 (0.466)
Acetate consumption	-0.03 (0.621)	0.004 (0.491)	0.165 (0.323)	-0.092 (5.71E-4)	-0.031 (0.0793)	-0.003 (0.731)	-0.114 (0.204)	-0.084 (0.669)	-0.11 (0.509)
Lactate consumption	0.041 (0.642)	-0.126 (8.76E-3)	0.214 (0.082)	-0.154 (0.014)	-0.177 (0.091)	-0.05 (0.029)	-0.08 (0.01)	0.042 (0.652)	-0.076 (0.596)
Propionate production	0.216 (0.133)	-0.229 (0.517)	-0.166 (0.204)	0.253 (1.5E-10)	0.065 (0.019)	0.158 (0.016)	0.227 (0.002)	-0.248 (0.06)	0.223 (0.039)
n-butyrate production	0.114 (0.693)	-0.193 (0.224)	0.139 (0.416)	0.12 (0.877)	0.028 (0.94)	0.041 (0.455)	0.03 (0.874)	-0.071 (0.653)	0.108 (0.683)
i-butyrate production	0.314 (0.003)	-0.17 (0.927)	-0.376 (0.08)	0.349 (0.115)	0.026 (0.343)	0.305 (4.41E-6)	0.067 (0.708)	-0.194 (0.852)	0.399 (5.53E-6)
n-valerate production	0.224 (0.223)	-0.208 (0.638)	-0.274 (0.048)	0.206 (0.353)	-0.196 (0.128)	0.307 (1.03E-4)	-0.088 (0.476)	-0.161 (0.225)	0.268 (0.013)
n-caproate production	-0.306 (0.029)	0.33 (0.252)	0.284 (0.12)	-0.423 (4.14E-4)	-0.305 (6.19E-4)	-0.234 (1.06E-3)	-0.149 (0.017)	0.362 (0.029)	-0.322 (7.02E-4)
n-caprylate production	-0.137 (0.653)	0.096 (0.396)	0.216 (0.897)	-0.09 (0.562)	-0.101 (0.571)	0.144 (0.73)	-0.003 (0.382)	0.077 (0.838)	-0.112 (0.441)
